# Dynamic Aberration Correction for Conformal Window of High-Speed Aircraft Using Optimized Model-Based Wavefront Sensorless Adaptive Optics

**DOI:** 10.3390/s16091414

**Published:** 2016-09-02

**Authors:** Bing Dong, Yan Li, Xin-li Han, Bin Hu

**Affiliations:** 1 School of Optoelectronics, Beijing Institute of Technology, Beijing 100081, China; hanoig@foxmail.com; 2 Beijing Institute of Space Mechanics & Electricity, Beijing 100090, China; 18610559376@163.com (Y.L.); 15210185191@139.com (B.H.)

**Keywords:** conformal optics, dynamic aberration, adaptive optics, wavefront sensorless, deformable mirror

## Abstract

For high-speed aircraft, a conformal window is used to optimize the aerodynamic performance. However, the local shape of the conformal window leads to large amounts of dynamic aberrations varying with look angle. In this paper, deformable mirror (DM) and model-based wavefront sensorless adaptive optics (WSLAO) are used for dynamic aberration correction of an infrared remote sensor equipped with a conformal window and scanning mirror. In model-based WSLAO, aberration is captured using Lukosz mode, and we use the low spatial frequency content of the image spectral density as the metric function. Simulations show that aberrations induced by the conformal window are dominated by some low-order Lukosz modes. To optimize the dynamic correction, we can only correct dominant Lukosz modes and the image size can be minimized to reduce the time required to compute the metric function. In our experiment, a 37-channel DM is used to mimic the dynamic aberration of conformal window with scanning rate of 10 degrees per second. A 52-channel DM is used for correction. For a 128 × 128 image, the mean value of image sharpness during dynamic correction is 1.436 × 10^−5^ in optimized correction and is 1.427 × 10^−5^ in un-optimized correction. We also demonstrated that model-based WSLAO can achieve convergence two times faster than traditional stochastic parallel gradient descent (SPGD) method.

## 1. Introduction

Currently, targeting and sensing on airborne platforms is mostly realized by imaging through a spherical or flat window. Although easy to design and fabricate, these windows show poor aerodynamic performance and can substantially increase air drag and radar cross section (RCS). For aircraft with high Mach number, an aspheric conformal window that follows the general contour of surrounding surface is preferred. However, the local shape of conformal windows changes across the field-of-regard (FOR), leading to large amounts of FOR-dependent wavefront aberrations and degraded images [1]. A variety of aberration correction methods using fixed or dynamic correctors have been proposed for conformal optics in recent years [2,3,4]. We are particularly interested in dynamic correction approach using a deformable mirror (DM) which can be taken as a freeform element in optical design [5]. Li et al. designed a missile seeker with an ellipsoidal conformal dome and a DM for correction [6,7]. The DM can be driven in open-loop or closed-loop mode. In open-loop mode, DM is shaped in response to parameters like look angle, window temperature and other flight conditions. The commands of DM for a given set of parameters are predetermined and stored in a lookup table, so a lot of simulations and experiments must be done to calibrate the control system before use. In closed-loop mode, DM is integrated into an adaptive optics (AO) control system [8]. The control signal of DM is derived from a dedicated wavefront sensor (i.e., classical AO) or from the final image detector (i.e., wavefront sensorless adaptive optics—WSLAO). Classical AO uses a wavefront sensor to measure aberration and a DM to make conjugated correction. The correction bandwidth of classical AO is usually higher than WSLAO. However, typical wavefront sensors like Hartmann-Shack may be inapplicable because of insufficient photons, non-common path error or structural constraints. A dedicated wavefront sensor is omitted in WSLAO and the control signal of DM is derived from the intensity information on the image detector. Employing WSLAO in an airborne imaging system can make most of the imaging photons and benefit from its simple structure.

The control algorithms of WSLAO can be divided into two categories: model-free algorithms and model-based algorithms. “Model-free” means the exact relationship between the metric function and aberration is unknown. Several model-free algorithms like stochastic parallel gradient descent (SPGD) have been proposed and demonstrated [9,10]. The common disadvantage of model-free algorithms is that a large number of iterations are usually needed and a global convergence is not always guaranteed. Several hundreds of iterations are typically needed to achieve convergence with SPGD, which severely limits its temporal bandwidth and dynamic correction performance. In model-based WSLAO, wavefront aberration is decomposed into specific modes like Zernike [11], Lukosz [12] or DM modes [13]. The choice of mode is based on the model used to describe the mathematical relationship between modal coefficients and metric function. The modal coefficients can be calculated directly from that relationship. The metric function can be derived from Strehl ratio (SR), image spectral density or mean-square spot radius [11,12,13]. Model-based WSLAO can achieve convergence much faster than model-free algorithms and avoid dropping into local optima, which are both crucial for dynamic aberration correction. The model-based WSLAO must be calibrated before correction in order for the DM to generate a specific mode, which is not required in model-free algorithm like SPGD. The influence functions of DM actuators should be measured by a wavefront sensor or interferometer in a calibration process.

In this paper, model-based WSLAO is applied for correction of dynamic aberration induced by conformal window of an infrared remote sensor on high-speed aircraft. Two approaches are proposed to accelerate the correction. Numerical simulations and experiments are made to demonstrate the feasibility and performance of our method.

## 2. Optical System

**Figure 1:**
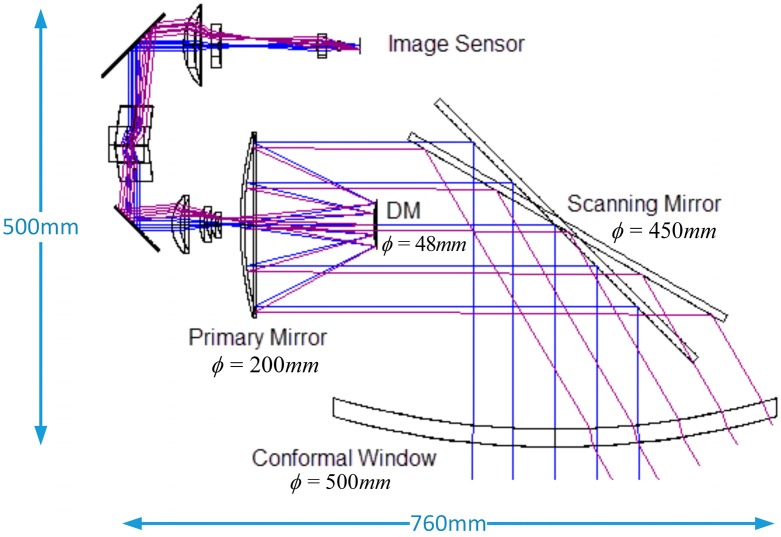
Optical system at 0° and 30° look angle.

The infrared remote sensing system coupled with a conformal window and a scanning mirror is depicted in Figure 1. The optical system is modeled and optimized in ZEMAX. The secondary mirror of the two-mirror reflective telescope is replaced by a DM that can be shaped to compensate the FOR-dependent aberrations. The system’s FOR is ±30° and the field of view (FOV) at any instant is ±0.25°. The conformal window is toroidal type that is formed by rotating a curve in the *Y*-*Z* plane about an axis parallel to *Y* axis. The working wavelength is in long-wave infrared from 7.7 to 10.3 μm. The system parameters are summarized in Table 1.

**Figure 2:**
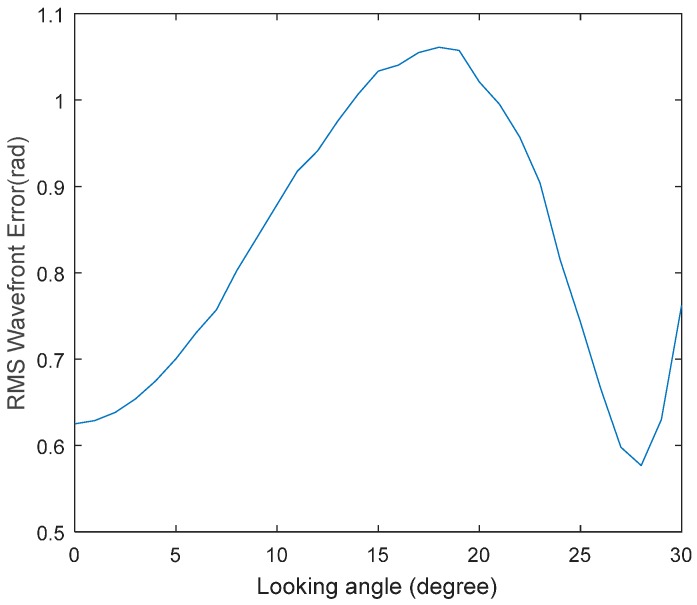
RMS of wavefront aberration as a function of look angle (at 9 μm).

**Figure 3:**
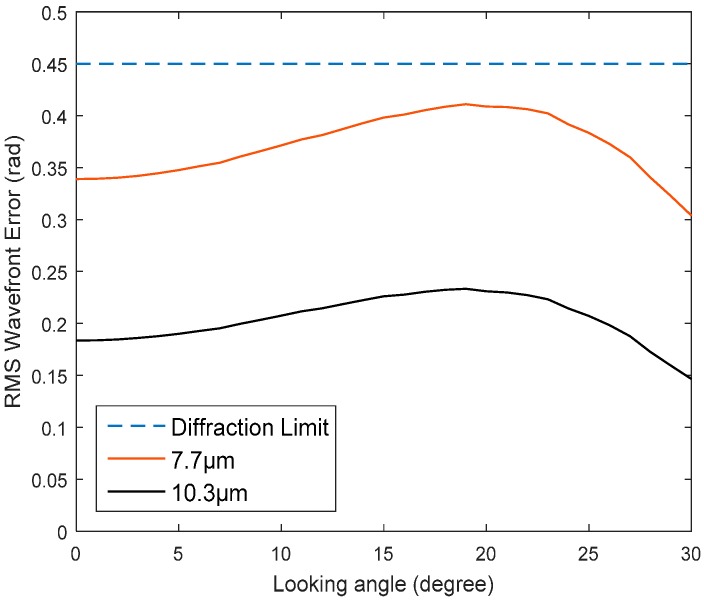
Residual error at 7.7 μm and 10.3 μm after correction at 9 μm.

The conformal window gives rise to aberrations that vary with look angle. The wavefront root-mean-square (RMS) value changing with look angle is shown in Figure 2. The window is symmetrical to the center of FOR, so we only show positive degrees. If we use an ideal DM to make an ideal wavefront correction at a wavelength of 9 μm, residual errors at other wavelengths are shown in Figure 3. The dotted line denoting diffraction limit corresponds to RMS error of 0.45 rad or Strehl Ratio of 0.8. Wavefront error lower than this criterion has little impact on image quality. So the system’s chromatic aberration which cannot be compensated by DM is negligible. In our simulations, the wavefront aberrations are estimated and corrected at 9 μm.

A critical parameter of DM is actuator stroke which might be insufficient in correction of large aberrations. As our system is working at infrared, the stroke requirement can be as large as 12 μm which is estimated from the peak-to-valley value of aberrations. Thanks to the development of DM technology, large-stroke DMs have been developed and are commercially available now [14,15,16].

## 3. Principle of Model-Based WSLAO

For extended objects, low spatial frequency content of the image spectral density *S_J_*(**m**) can be used as a metric function in model-based WSLAO:

(1) $$G={\left[\int_{0}^{2\pi }\int_{M_{1}}^{M_{2}}S_{J}(m)mdmd\xi \right]}^{-1}$$

where M_1_ and M_2_ are normalized spatial frequency, *ξ* is the angle of spatial frequency, and **m** = (*m*cos*ξ*, *m*sin*ξ*) is the spatial frequency vector. The relationship between wavefront aberration **Φ** and the metric function *G* is given by:

(2) $$G\approx q_{1}+q_{2}\frac{1}{\pi }\iint_{p}{\left|\nabla \Phi \right|}^{2}ds$$

where *q*_1_ and *q*_2_ are constants depending on object structure. $\iint_{p}ds$ denotes an integral over the pupil area *p*. Wavefront aberration **Φ** can be represented by a series of Lukosz modes *L_i_* whose derivatives are orthogonal with each other:

(3) $$\Phi =\sum_{i=4}^{N}a_{i}L_{i}$$

(4) $$\iint_{p}\nabla L_{i}\cdot \nabla L_{j}ds=\{\begin{array}{cc} 1 & i=j\\ 0 & i\neq j \end{array}$$

The expression and aberration definition of Lukosz modes can be found in [12]. Using Equations (3) and (4), the metric function can be rewritten in terms of Lukosz coefficients:

(5) $$G=q_{1}+q_{2}\sum_{i=4}^{N}{a_{i}}^{2}$$

The initial metric function is assumed as *G*_0_. After introducing a positive modal bias +*b_i_L_i_* by DM, the metric function becomes *G*_+_. Applying a negative modal bias –*b_i_L_i_* corresponds to metric function of *G*_–_. Then we can get an equation set as:

(6) $$\{\begin{array}{l} G_{0}=q_{1}+q_{2}\sum_{k\neq i}{a_{k}}^{2}+q_{2}{a_{i}}^{2}\\ G_{+}=q_{1}+q_{2}\sum_{k\neq i}{a_{k}}^{2}+q_{2}{\left(a_{i}+b_{i}\right)}^{2}\\ G_{-}=q_{1}+q_{2}\sum_{k\neq i}{a_{k}}^{2}+q_{2}{\left(a_{i}-b_{i}\right)}^{2} \end{array}$$

The required correction amount for each mode can be estimated by solving Equation (6):

(7) $$a_{i,\mathrm{corr}}=-a_{i}=-\frac{b_{i}(G_{+}-G_{-})}{2G_{+}-4G_{0}+2G_{-}}$$

**Figure 4:**
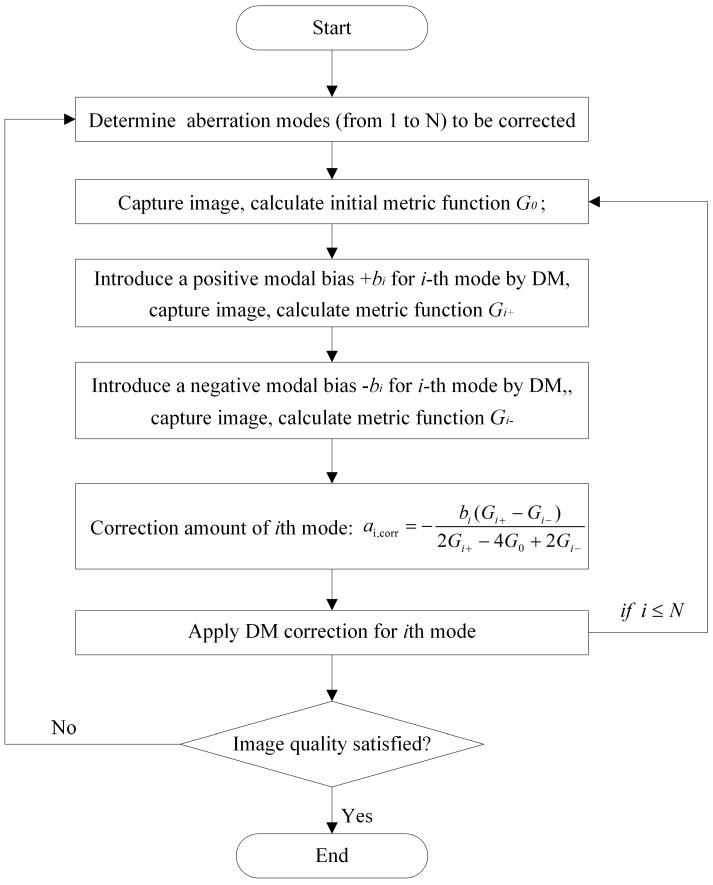
Algorithm flow chart of 3N algorithm.

From Equation (7), to estimate one modal coefficient, at least three metric function measurements are required. To correct N modes, two strategies might be used. One can apply correction for all modes simultaneously after their coefficients are estimated, which requires 2N + 1 images in total. Alternatively, the correction can be applied in turn for each mode immediately after its coefficient is estimated. This strategy requires 3N images in total as the metric function with zero modal bias (i.e., *G*_0_) must be recalculated for each mode. The flow chart of 3N algorithm is shown is Figure 4. We will compare the dynamic correction performance of these two algorithms in next sections.

## 4. Algorithm Optimization and Simulation

### 4.1. Simulation Method

The model-based algorithm is programmed in the MATLAB environment. The wavefront aberration induced by conformal window shape at any look angle is obtained from the optical design software (ZEMAX). These aberrations are extracted into MATLAB through its dynamic data exchange (DDE) interface with ZEMAX. The compensated DM deformation calculated by model-based algorithm is sent back to ZEMAX to evaluate the image quality after correction.

Assuming that sampling width of the object is L and diameter of the pupil is D, then the sampling interval of spatial frequency is equal to 1/L. To improve the sampling rate and prevent the alias effect, L = γD (γ > 1) is usually adopted by padding zero outside the pupil. Here γ is the zero-padding factor which corresponds to the number of samples along radial direction within λ/D at the image plane. The wavefront aberration for any field of view obtained from ZEMAX is sampled by a 128 × 128 grid and then embedded to a 256 × 256 matrix (i.e., γ = 2). The extended object is chosen as a typical remote sensing image with 256 × 256 pixels. The detected image is generated by two-dimensional discrete convolution of object and the point spread function. As proven in [12], the wavefront estimation of our algorithm is independent of object structure.

**Figure 5:**
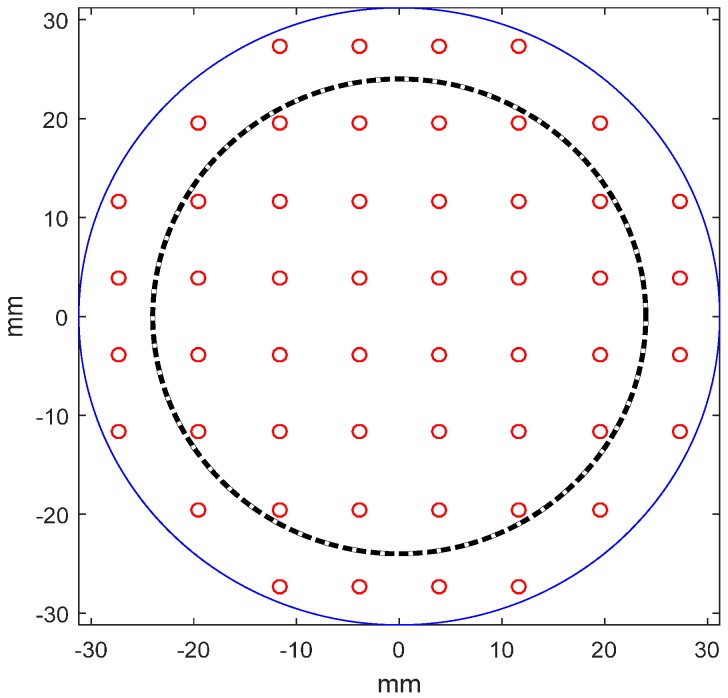
Layout of DM with 52 actuators.

The layout of DM with 52 actuators (red circles) is depicted in Figure 5. All influence functions are assumed identical. However, not all actuators are fully illuminated. The dashed inner-circle in Figure 5 denotes the clear aperture of DM. The influence function is assumed to be Gaussian-type as given in Equation (8). The coupling coefficient of DM with continuous faceplate is usually between 10% and 20%. Here the coupling coefficient *c* is set as 0.2. The actuator stroke is larger than 12 μm as estimated in Section 2:

(8) $$I_{i}(x,y)=\exp\left[\ln c\frac{{\left(x-x_{i}\right)}^{2}+{\left(y-y_{i}\right)}^{2}}{d^{2}}\right]$$

Here$\left(x_{i},y_{i}\right)$ denotes actuator position and d is actuator pitch.

### 4.2. Optimized Dynamic Correction

For dynamic aberration correction, the temporal bandwidth of the AO system must be higher than that of aberration. Besides using more powerful hardware (i.e., image detector and DM with high frame rate) [17], the wavefront control algorithm must also be optimized to accelerate the convergence. Two approaches are investigated here to improve the efficiency of the model-based algorithm. The first one is to reduce the mode number used in correction. As shown in Equation (7), the estimation of modal coefficient is independent of each other. If we have a priori knowledge of the aberration, making corrections only on those dominant modes with large amplitude can significantly improve the correction speed.

**Figure 6:**
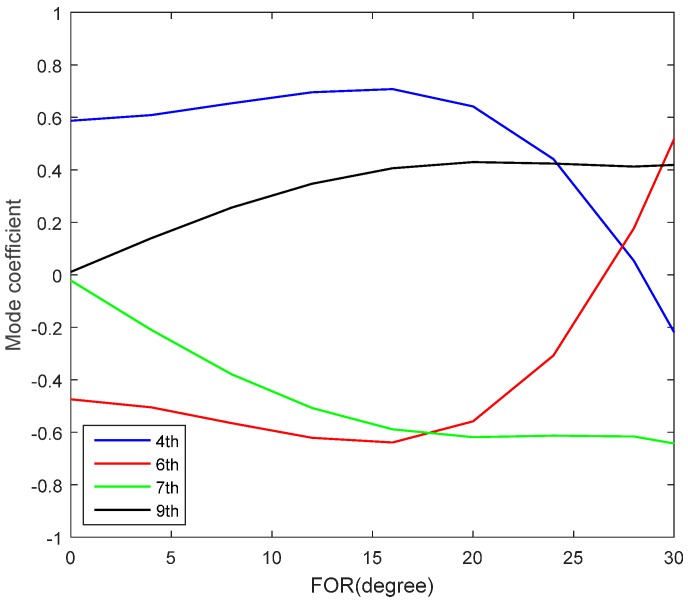
Coefficients of dominant Lukosz modes varying across FOR.

**Figure 7:**
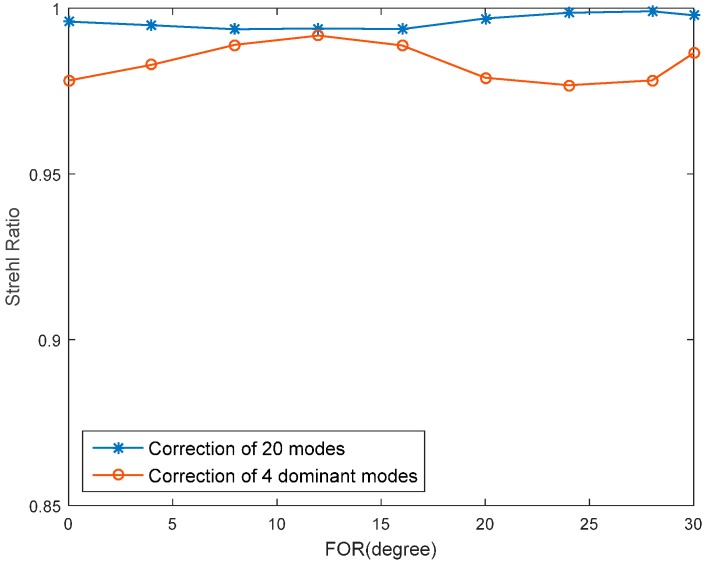
Strehl ratio variation curve in correction of first 20 modes or four dominant modes.

The wavefront aberrations induced by conformal window and subsequent imaging system can be obtained from ZEMAX. Then the aberrations are decomposed into Lukosz modes. The dominant Lukosz modes of our system are 4th (defocus), 6th (astigmatism), 7th (coma), 9th (trefoil) and their coefficients varied across the FOR are shown in Figure 6. The coefficients of other modes are less than 0.1. The modal coefficient used here is normalized by its RMS value, which means a unit amplitude of mode corresponding to a phase aberration with RMS of 1 rad. The correction results of four dominant Lukosz modes and first 20 Lukosz modes are both shown in Figure 7 for comparison. The Strehl ratio after correction of 20 modes is just slightly better than correction of 4 dominant modes. However, 32 more images have to be captured and then evaluated when using “2N + 1” algorithm. Here Strehl ratio is calculated from the RMS (*σ*) of wavefront aberration by exp(–*σ*^2^) [18].

In practice, wavefront aberrations may not only arise from the asymmetry of the conformal window but also be induced by aero-optical effects. The advantage of using DM on-board is that the mirror shape can be adjusted accordingly to accommodate various disturbances. The aberrations caused by aero-optical effects are random and much more complicated than conformal window shape. The magnitude and distribution of aero-optical aberrations depends on many factors such as flight speed and attitude, thermal environment, window shape and material, etc. [19]. However, it is still possible to predict the aero-optical aberrations by simulation and experiment and then extract dominant aberration modes, which is our future work [20,21]. Alternatively, low order DM modes can be selected for correction if we have no prior knowledge of the aberration [13].

The second approach to accelerate the correction is to minimize the image size while keeping satisfactory corrected accuracy. The most time-consuming part of our algorithm is the image acquisition process and subsequent calculation of metric function that involves two-dimensional Fast Fourier Transform (FFT) of captured image. The computing complexity of FFT is *O* (*M* log *M*) where M is the image size, so using a smaller image can save a lot of computational time thus improve the correction speed. For point-like targets, low order modes only affect the intensity near the center of image spots. In this paper, we use extended targets and the metric function is low spatial frequency content of the image spectral density. Adopting a smaller image leads to a larger sampling interval in the spatial frequency domain and reduces the correction accuracy. Simulations can be made to determine the minimum value of image size.

**Figure 8:**
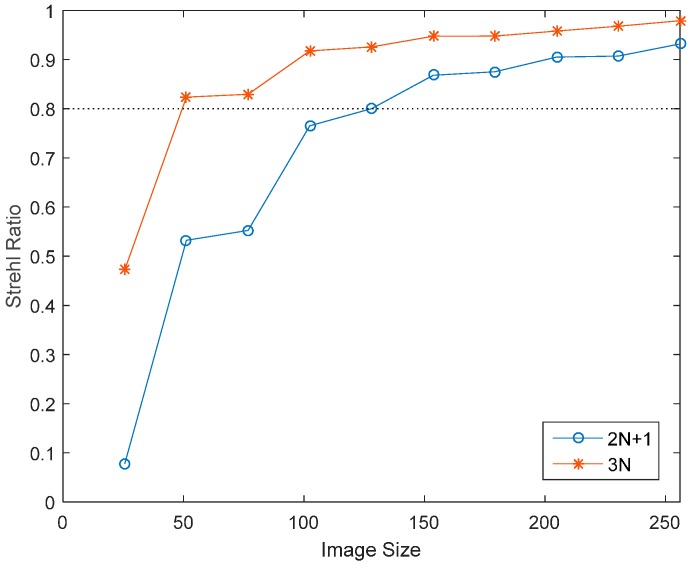
Strehl ratio variation with image size.

For 100 samples of random aberrations composed of first 15 Lukosz modes with RMS of 1 rad, the mean Strehl ratio after correction is calculated for different image size in Figure 8. The correction results of using 2N + 1 images and 3N images (here mode number N = 15) are both given for comparison. To obtain Strehl ratio better than 0.8, images containing at least 50 × 50 pixels are required for “3N algorithm” and at least 150 × 150 pixels for “2N + 1 algorithm”. From Figure 8, “3N algorithm” is superior to “2N + 1 algorithms” in correction accuracy for same image size. In “3N algorithm”, each modal aberration is corrected by DM immediately after the estimation of each modal coefficient, which is more appropriate for fast-changing dynamic aberration and can alleviate the crosstalk problem in coefficient estimation.

### 4.3. Dynamic Correction Simulation

In our application, the speed at which aberrations change depends on the mirror scan rate, without consideration of aero-optical effects. In practice, the scan rate depends on many factors like the aircraft’s speed and height, frame rate of the detector, radiation intensity of observed targets and the speed of the data processing unit. The scan rate may vary widely during the flight mission, from several to tens of degrees per second. What we care about most is the AO system’s performance under different scan rates. Because of the constantly changing aberration and inevitable time delay of AO systems, DM can only create a complementary wavefront for the current look angle based on images acquired at previous look angles. The time delay in each correction cycle, typically on the order of milliseconds level, depends on the specific correction algorithm and AO hardware configuration. However, partial correction is still possible if the aberration changing is continuous with no abrupt disturbance.

**Figure 9:**
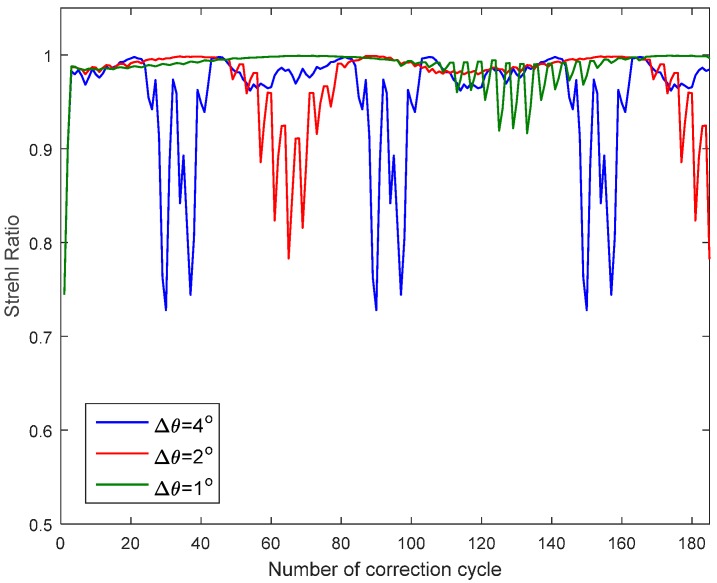
Strehl ratio variation in dynamic correction for different scanning rate.

To predict the dynamic performance of AO, we do not need to know the exact time delay value and the exact scanning rate. We only need to know how much the aberration changed after the time delay. The time delay for estimation and correction of a single mode (i.e., one correction cycle) is assumed as t seconds which includes time of image acquisition, metric function calculation, DM response. After t seconds, the change in look angle is assumed as Δθ. If Δθ is small, the correction is still effective because the aberration changing is usually continuous without abrupt disturbance. Based on the DDE interface between MATLAB and ZEMAX, we can get dynamic correction result for any given Δθ. Given Δθ as 1°, 2° and 4°, the Strehl ratio after each correction cycle is shown in Figure 9. From Figure 9, the variation of look angle during time t should be less than 2° to keep Strehl ratio better than 0.8. The rapid fluctuation region of Strehl ratio curve is caused by the fast change of the aberration. From Figure 2, this region roughly corresponds to look angle from 25° to 30°.

## 5. Experimental Demonstration

### 5.1. System Setup

**Figure 10:**
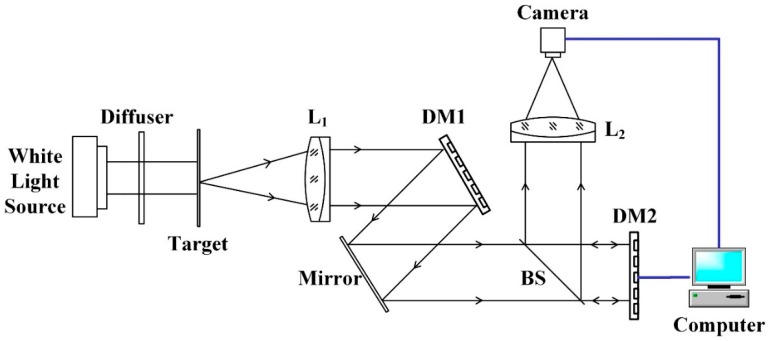
Experimental system layout.

Although the optical system in Section 2 is working in the infraredregion, the algorithm can be demonstrated in the visible range. The experimental system is depicted in Figure 10. The observation target illuminated by a white light source is a remote sensing image printed on a transparent film. DM1, a 37-channel micromachined membrane DM, is used to mimic dynamic aberrations induced by conformal window. The mirror’s scanning rate can be reflected by the refresh rate of DM1. DM2, a large-stroke DM with 52 electromagnetic actuators, is used for wavefront correction as well as providing appropriate modal bias in aberration estimation. The target is finally imaged by a 12-bit charge-coupled device (CCD) camera. The camera and DM2 are both connected to the computer to form a closed-loop control system.

### 5.2. System Error Clearance

**Figure 11:**
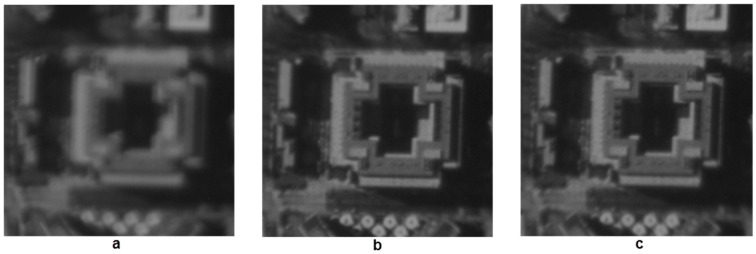
(**a**) Image with initial aberration, J = 1.385 × 10^−5^; (**b**) Image after SPGD correction, J = 1.459 × 10^−5^; (**c**) Image after model-based WSLAO correction, J = 1.457 × 10^−5^. Image size: 128 × 128.

**Figure 12:**
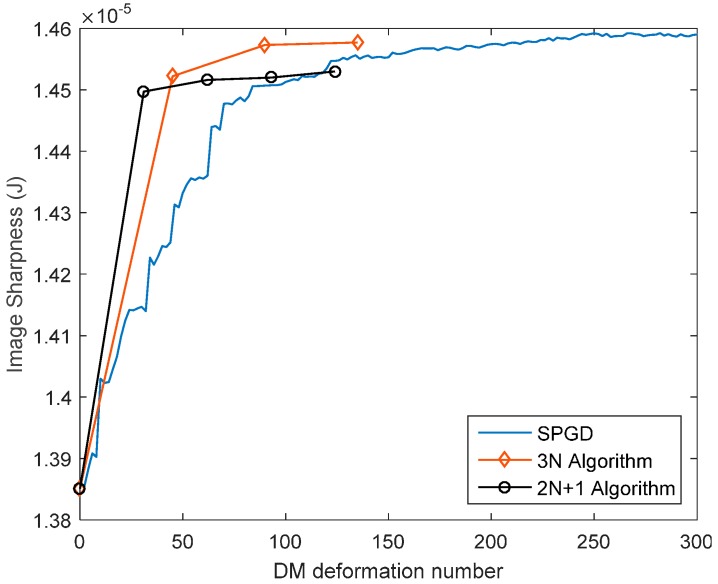
Image sharpness vs. DM deformation number in SPGD and model-based WSLAO.

The initial system errors induced by figure error of DMs and misalignment should be removed by DM itself before adding any specific aberrations. Both model-free and model-based WSLAO were used to calibrate the system and the results are shown in Figure 11. The model-free algorithm used here is SPGD and image sharpness defined by Equation 9 is used as its metric function. In model-based algorithm, first 15 Lukosz modes are estimated and corrected using “3N algorithm”. The variation of image sharpness in SPGD and model-based algorithm are shown in Figure 12:

(9) $$J=\frac{\iint I^{2}(x,y)dxdy}{{\left(\sum I(x,y)\right)}^{2}}$$

where I (x, y) is the intensity distribution at image plane.

From Figure 11 and Figure 12, the correction result of SPGD is slightly better than that of the model-based WSLAO, however, at the cost of more DM deformation number and more computation time. From Figure 12, the image sharpness in the 3N algorithm increases from 1.385 × 10^−5^ to 1.457 × 10^−5^ after 90 DM deformations. However, 200 DM deformations are required in SPGD to achieve the same improvement, so the convergence speed of the -based WSLAO (3N algorithm) is roughly two times faster than SPGD. In Figure 12, we use the DM deformation number to evaluate the convergence speed instead of using iteration number which can be ambiguous for different algorithms. In model-based WSLAO, DM is deformed 2N + 1 or 3N times in one iteration. However, in SPGD, DM is deformed only twice in one iteration. The correction accuracy of “3N” algorithm is also higher than “2N + 1” algorithm from Figure 12.

### 5.3. Dynamic Correction Results

**Figure 13:**
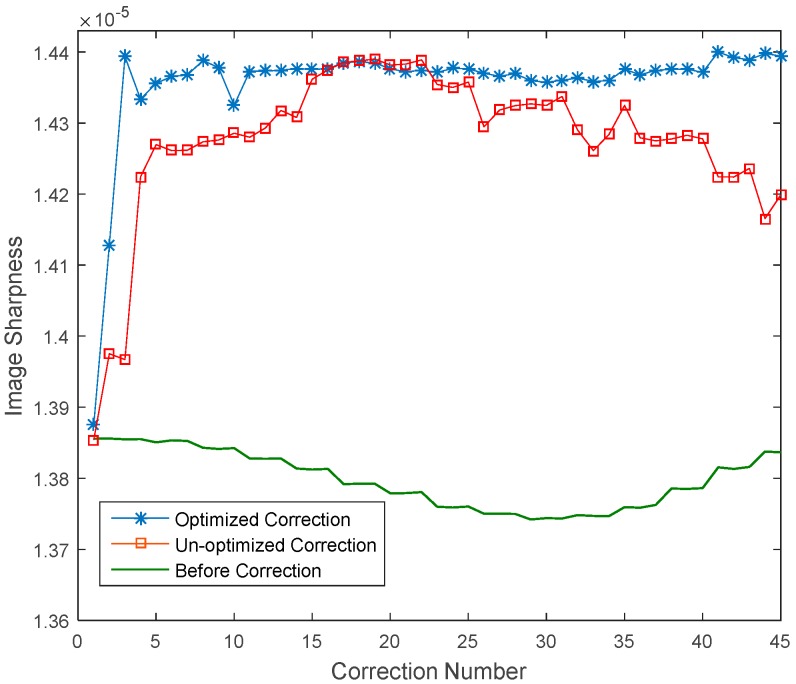
Dynamic correction results using the optimized method and the unoptimized method.

After removing the system’s initial error, dynamic aberrations of the conformal window across full FOR as given in Section 2 are sampled with 1 degree interval and then reproduced by DM1 with update rate of 10 Hz, corresponding to a scanning rate of 10 degrees per second. The time delay in correction of one mode is about 90 milliseconds, which is limited by our image acquisition hardware. DM2 is used to compensate for the aberration and the correction results are shown in Figure 13. Here the “3N” algorithm is used and the image sharpness as defined in Equation (9) is used to evaluate the image quality after correction of each mode. Dominant Lukosz modes as shown in Figure 6 are corrected in optimized correction. First 15 Lukosz modes are corrected in un-optimized correction. Images are captured with the same size of 128 × 128 as a tradeoff between correction speed and accuracy. From Figure 13, using the optimized method can achieve convergence much faster than un-optimized method. The mean value of image sharpness during dynamic correction is 1.436 × 10^−5^ in optimized correction and is 1.427 × 10^−5^ in unoptimized correction.

## 6. Conclusions

An infrared remote sensing system with conformal window and secondary deformable mirror was fully established. Model-based WSLAO was proposed and optimized for dynamic correction of aberrations induced by conformal window. To optimize the correction, only some dominant Lukosz modes are corrected and the image size is minimized to save the computing time of metric function. In experiment, dynamic aberration of conformal window with scanning rate of 10 degrees per second is reproduced by a 37-channel DM. A 52-channel DM is used for correction. For a 128 × 128 image, the mean value of image sharpness during dynamic correction is 1.436 × 10^−5^ in optimized correction and is 1.427 × 10^−5^ in un-optimized correction. We also demonstrated that the model-based WSLAO can achieve convergence two times faster than traditional stochastic parallel gradient descent (SPGD) method.

Dynamic aberrations caused by aero-optical effects is not taken into account in this paper. Testing the performance of model-based WSLAO in correction of aero-optical aberration will be the subject of our future work. The temporal bandwidth of aero-optical aberrations surrounding the conformal window can be much higher than that of dynamic aberrations induced by window shape and scanning mirror. The temporal bandwidth of aero-optical aberrations is on the order of KHz for boundary layer turbulence. It is much more challenging to compensate aero-optical aberrations by WSLAO system. The wavefront control algorithm must be optimized and more powerful hardware should be used. To accelerate the correction of model-based WSLAO, dominant aberration modes induced by aero-optical effects should be determined in advance by simulation or experiment. Micro-electro-mechanical-system (MEMS) DM with high response speed (up to 50 KHz) [22] can be used to further decrease the time delay.

## Figures and Tables

**Table 1:** System Parameters.

Parameter	Value	Parameter	Value
Window surface type	Toroid	Window thickness (mm)	20
Window material	Germanium	Window diameter (mm)	500
Field of view (°)	±0.25	Field of regard (°)	±30
Primary mirror diameter (mm)	200	DM diameter (mm)	48
Working f-number	2.8	Working wavelength (μm)	7.7–10.3
